# Four mutations in *MITF*, *SOX10* and *PAX3* genes were identified as genetic causes of waardenburg syndrome in four unrelated Iranian patients: case report

**DOI:** 10.1186/s12887-021-02521-6

**Published:** 2021-02-08

**Authors:** Safoura Zardadi, Sima Rayat, Maryam Hassani Doabsari, Aliagha Alishiri, Mohammad Keramatipour, Zeynab Javanfekr Shahri, Saeid Morovvati

**Affiliations:** 1 Department of Biology, School of Basic Sciences, Science and Research Branch, Islamic Azad University, Tehran, Iran; 2 Tehran Medical Sciences, Islamic Azad University, Tehran, Iran; 3 Faculty of Medicine, Hormozgan University of Medical Sciences, Hormozgan, Iran; 4 Department of Medical Genetics, Faculty of Medicine, Tehran University of Medical Sciences, Tehran, Iran; 5 School of Advanced Sciences and Technology, Islamic Azad University-Tehran Medical Sciences, Tehran, Iran; 6 Department of Genetics, Faculty of Advanced Sciences and Technology, Tehran Medical Sciences, Islamic Azad University, Tehran, Iran

**Keywords:** Waardenburg syndrome, *MITF*, *SOX10*, *PAX3*

## Abstract

**Background:**

Waardenburg syndrome (WS) is a rare genetic disorder. The purpose of this study was to investigate clinical and molecular characteristics of WS in four probands from four different Iranian families.

**Case presentation:**

The first patient was a 1-year-old symptomatic boy with congenital hearing loss and heterochromia iridis with a blue segment in his left iris. The second case was a 1.5-year-old symptomatic girl who manifested congenital profound hearing loss, brilliant blue eyes, and skin hypopigmentation on the abdominal region at birth time. The third patient was an 8-month-old symptomatic boy with developmental delay, mild atrophy, hypotonia, brilliant blue eyes, skin hypopigmentation on her hand and foot, Hirschsprung disease, and congenital profound hearing loss; the fourth patient was a 4-year-old symptomatic boy who showed dystopia canthorum, broad nasal root, synophrys, skin hypopigmentation on her hand and abdomen, brilliant blue eyes, and congenital profound hearing loss. Whole exome sequencing (WES) was used for each proband to identify the underlying genetic factor. Sanger sequencing was performed for validation of the identified mutations in probands and the available family members. A novel heterozygous frameshift mutation, c.996delT (p.K334Sfs*15), on exon 8 of the *MITF* gene was identified in the patient of the first family diagnosed with WS2A. Two novel *de novo* heterozygous mutations including a missense mutation, c.950G > A (p.R317K), on exon 8 of the *MITF* gene, and a frameshift mutation, c.684delC (p.E229Sfs*57), on the exon 3 of the *SOX10* gene were detected in patients of the second and third families with WS2A and PCWH (Peripheral demyelinating neuropathy, Central dysmyelinating leukodystrophy, Waardenburg syndrome, Hirschsprung disease), respectively. A previously reported heterozygous frameshift mutation, c.1024_1040del AGCACGATTCCTTCCAA, (p.S342Pfs*62), on exon 7 of the *PAX3* gene was identified in the patient of the fourth family with WS1.

**Conclusions:**

An exact description of the mutations responsible for WS provides useful information to explain the molecular cause of clinical features of WS and contributes to better genetic counseling of WS patients and their families.

## Background

Waardenburg syndrome (WS) is an auditory-pigmentary syndrome with an estimated prevalence of approximately 1:40,000 in the general population; it is responsible for 2–5 % of congenital deafness in total [1, 2]. The disorder is characterized by varying degrees of sensorineural hearing loss and pigmentary abnormalities of the hair, skin, and eye. The severity of such congenital hearing loss varies within/between families, ranging from mild to profound, and is usually non-progressive; it can also be either unilateral or bilateral while bilateral is more common [3–5]. Pigmentary changes of iris in individuals with WS include complete heterochromia iridum that each eye is a different color, partial or segmental heterochromia that segments of blue or brown pigmentation exist in one or both eyes and characteristic brilliant blue in both eyes. Pigmentary disturbances of the hair include white forelock which is observed in at least one-third of individuals with WS type 1 (WS1) or WS type 2 (WS2) and usually involves the forehead but may occur elsewhere; premature greying before age 30 and white hairs can also be evident within eyelashes, eyebrow, or at other sites on the body. On top of that, skin hypopigmentation may be found on the face, trunk, or limbs [4, 6–9].

Depending on the presence or absence of additional symptoms and genetic criteria, WS is divided into four subtypes as in WS1 to WS4. WS1 (OMIM: 193,500) and WS2 (OMIM: 193,510) are the most common types, while WS3 (OMIM: 148,820) and WS4 (OMIM: 277,580) are not the common types [10–14].

WS1 is an autosomal dominant disorder that is caused by mutations within the gene paired box gene 3 (*PAX3*) on chromosome 2q36.1. WS1 is phenotypically similar to WS2, but the presence of dystopia canthorum -a prominent feature of WS1 with a penetrance of 97 %- is specific for WS1. Sensorineural hearing loss is more common in WS2 than WS1 with an incidence rate of 60 % in WS1 to 90 % in WS2 [5, 11, 15–17].

WS2 is a heterogeneous disorder and is categorized into five subtypes based on the genetic causes. Approximately, 15 % of WS2 cases have mutations in the microphthalmia-associated transcription factor (*MITF*) gene on chromosome 3p14.1-p12.3, termed WS2A (OMIM: 193,510). Mutations in SRY (sex-determining region Y)-box10 (*SOX10)* gene on chromosome 22q13.1 are associated with WS2E (OMIM: 611,584) which occurs in about 15 % of WS2 and also mutations in the snail homolog of 2 (*SNAI2*) gene, mapped to chromosome 8q11.21, result in WS2D (OMIM: 608,890). Two loci embracing WS2B (OMIM: 600,193) and WS2C (OMIM: 606,662) have been localized on chromosome 1p21-p13.3 and 8p23, respectively [12, 16]. Also, occasional cases with WS2 are associated with mutations in endothelin-3 (*EDN3)* and endothelin receptor type B *(EDNRB)* genes. WS2 is mainly inherited in an autosomal dominant manner, but some cases of WS2 are caused by recessive mutations in the *SNAI2* gene. More than half of WS2 patients are not explained at the molecular level [5, 14, 18, 19].

WS3 or Klein-Waardenburg syndrome -the severe presentation of WS1- with the autosomal dominant mode of inheritance is caused by heterozygous or homozygous mutations of the *PAX3* gene. WS3 is characterized by the presence of musculoskeletal abnormalities of limbs (syndactyly, joint contractures, and muscle hypoplasia) in addition to the common phenotypes of WS1 [17, 20].

WS4 or Shah-Waardenburg syndrome is characterized by the association of Hirschsprung disease in addition to clinical features of WS2. Hirschsprung disease (OMIM: 142,623) is characterized by the presence of aganglionic megacolon that in turn is caused by the congenital deficiency of neural crest from enteric ganglia. Three disease-causing genes are involved in the pathogenesis of WS4; mutations in *EDNRB* gene on chromosome 13q22.3 can cause WS4A. Mutations of the *EDN3* gene on chromosome 20q13.2-q13.3 have been identified in patients with WS4B. Around 50 % of WS4 patients are justifiable due to *SOX10* mutations that are dominantly inherited and are called ‘WS4C’ while 20–30 % of WS4 patients are due to *EDNRB* and *EDN3* mutations that are inherited in an autosomal recessive pattern. In a nutshell, a minority of WS4 patients cannot be identified molecularly [12, 14, 16, 17, 21, 22].

Though many mutations in *MITF*, *SOX10*, and *PAX3* genes associated with WS2A, PCWH, and WS1 have been identified respectively in different ethnic groups, there is little to no studies in Iranian populations; thus this study is the first report of a genetically diagnosed case of PCWH in Iranian population. Herein, we aimed to investigate the clinical features and genotypes of four unrelated Iranian patients with WS. In sum, we identified two novel heterozygous mutations in the *MITF* gene in patients manifesting WS2A, a novel heterozygous mutation in the *SOX10* gene in an affected individual with PCWH, and a reported heterozygous mutation in the *PAX3* gene in a WS1 patient [5, 23–31].

## Case presentation

**Fig. 1:**
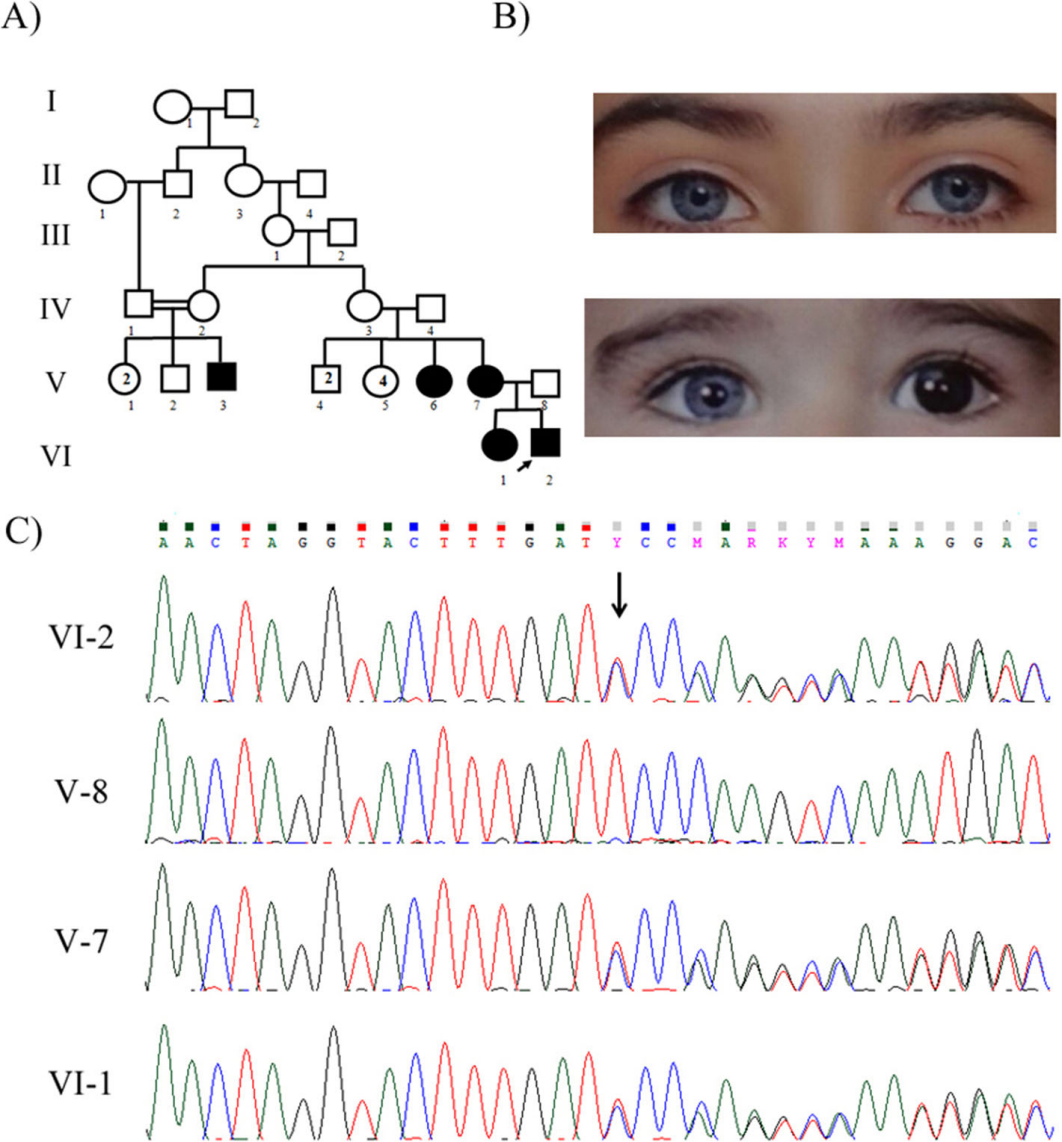
**a** Pedigree of family 1 with Waardenburg syndrome type 2A with *MITF* mutation. The black arrow indicates the proband (VI-2). **b** Iris color of Waardenburg syndrome type 2 patients. VI-1, brilliant blue irises. VI-2, heterochromia iridis, and a blue segment in his left iris. **c** The DNA sequence chromatogram of exon 8 of the *MITF* gene with the frameshift mutation c.996delT (p.K334Sfs*15) in heterozygous state in proband )VI-2), proband^’^s mother (V-7), and proband^’^s sister (VI-1). This mutation was not found in proband^’^s father (V-8). The black arrow above the chromatogram sequence shows the c.996delT mutation

**Fig. 2:**
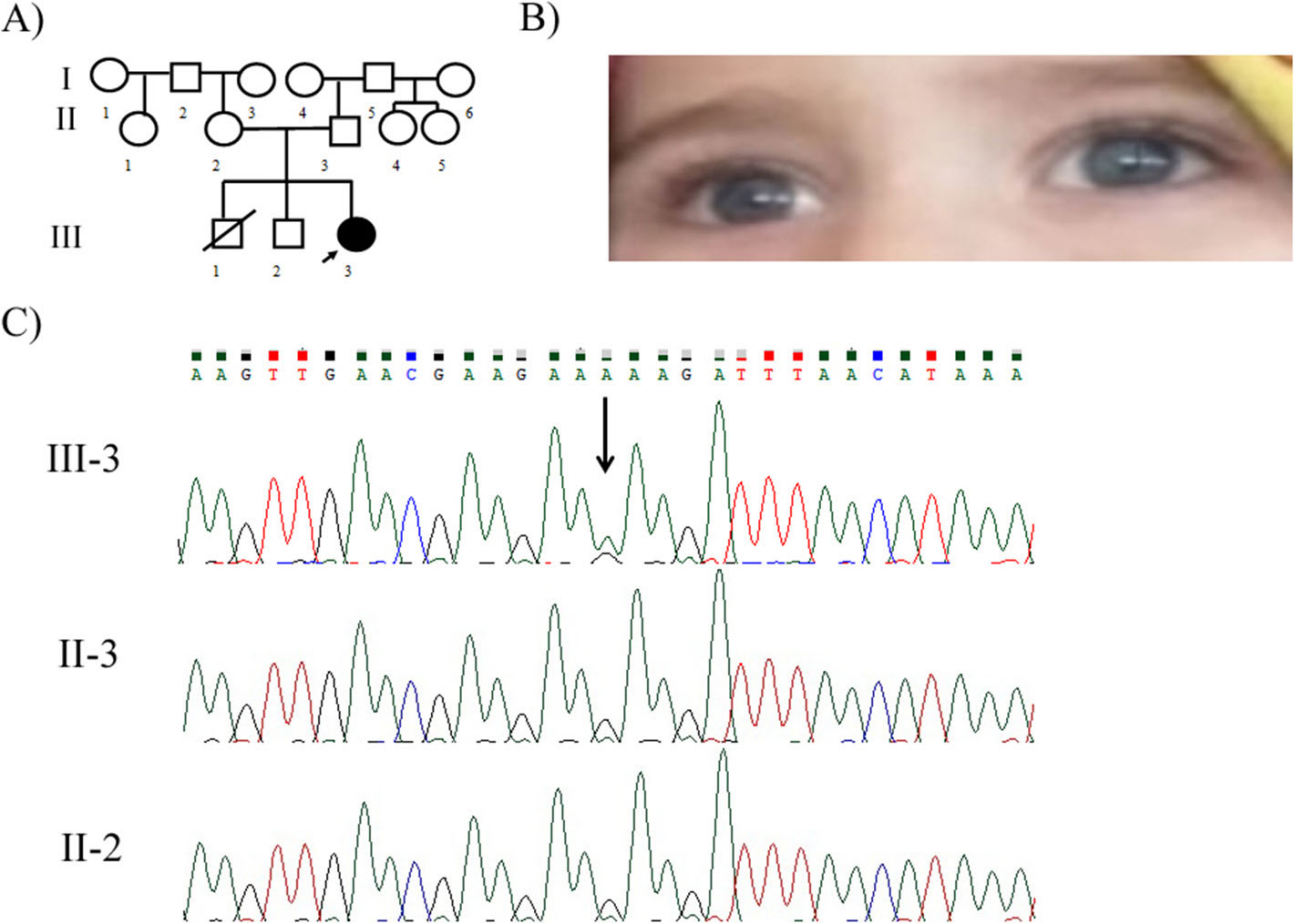
**a** Pedigree of family 2 with Waardenburg syndrome type 2A with *MITF* mutation. The black arrow indicates the proband (III-3). **b** Iris color of Waardenburg syndrome type 2 patient. III-3, brilliant blue irises. **c** The DNA sequence chromatogram of exon 8 of the *MITF* gene with the missense mutation c.950G > A (p.R317K) in heterozygous state in proband )III-3). This mutation was not detected in proband^’^s father (II-3) and proband^’^s mother (II-2). The black arrow above the chromatogram sequence shows the c.950G > A mutation

**Fig. 3:**
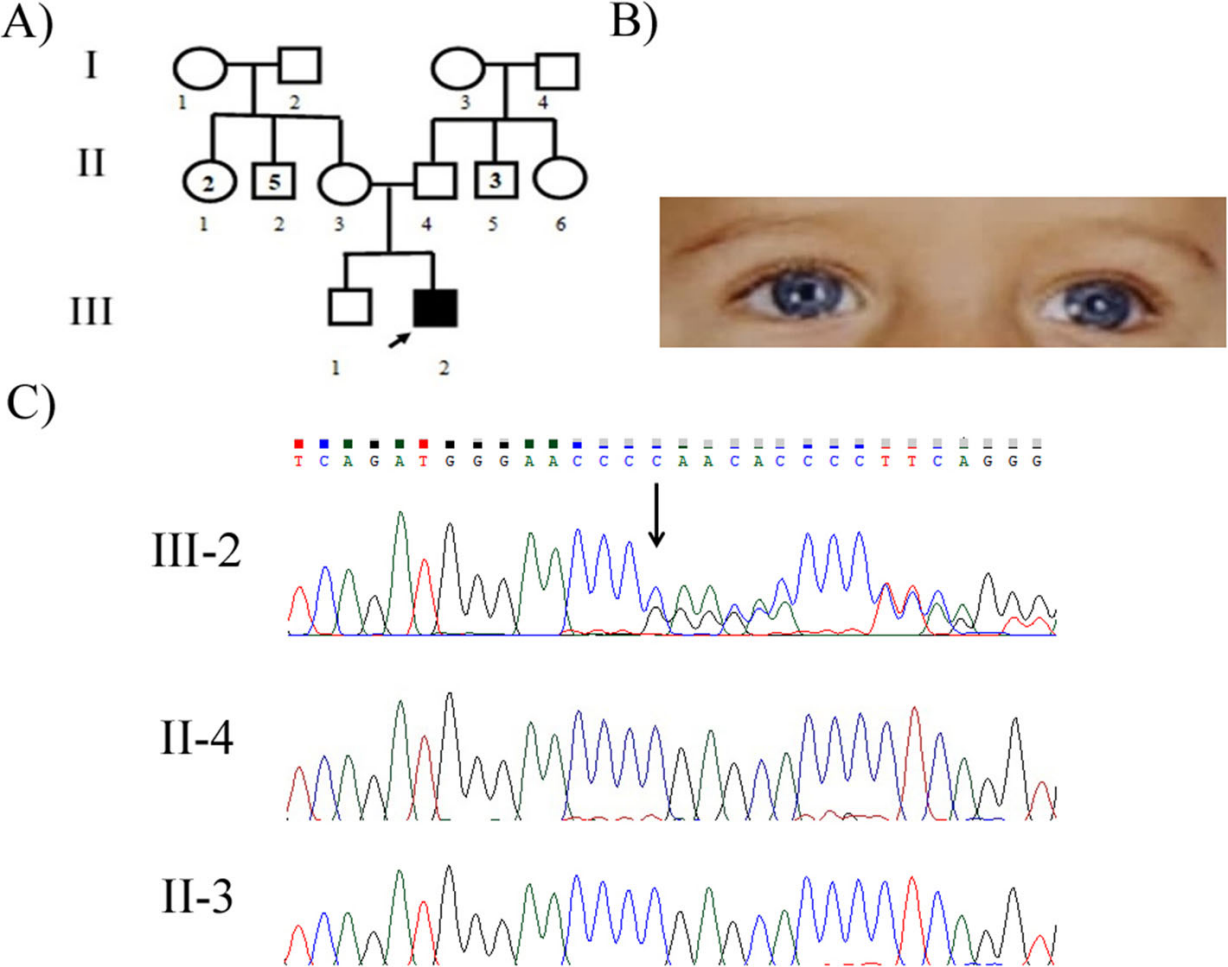
**a** Pedigree of family 3 with PCWH (Peripheral demyelinating neuropathy, Central dysmyelinating leukodystrophy, Waardenburg syndrome, Hirschsprung disease) with *SOX10* mutation. The black arrow indicates the proband (III-2). **b** Iris color of PCWH patient. III-2, brilliant blue irises. c The DNA sequence chromatogram of exon 3 of the *SOX10* gene with the frameshift mutation c.684delC (p.E229Sfs*57) in heterozygous state in proband )III-2). This mutation was not observed in proband^’^s father (II-4) and proband^’^s mother (II-3). The black arrow above the chromatogram sequence shows the c.684delC mutation

**Fig. 4:**
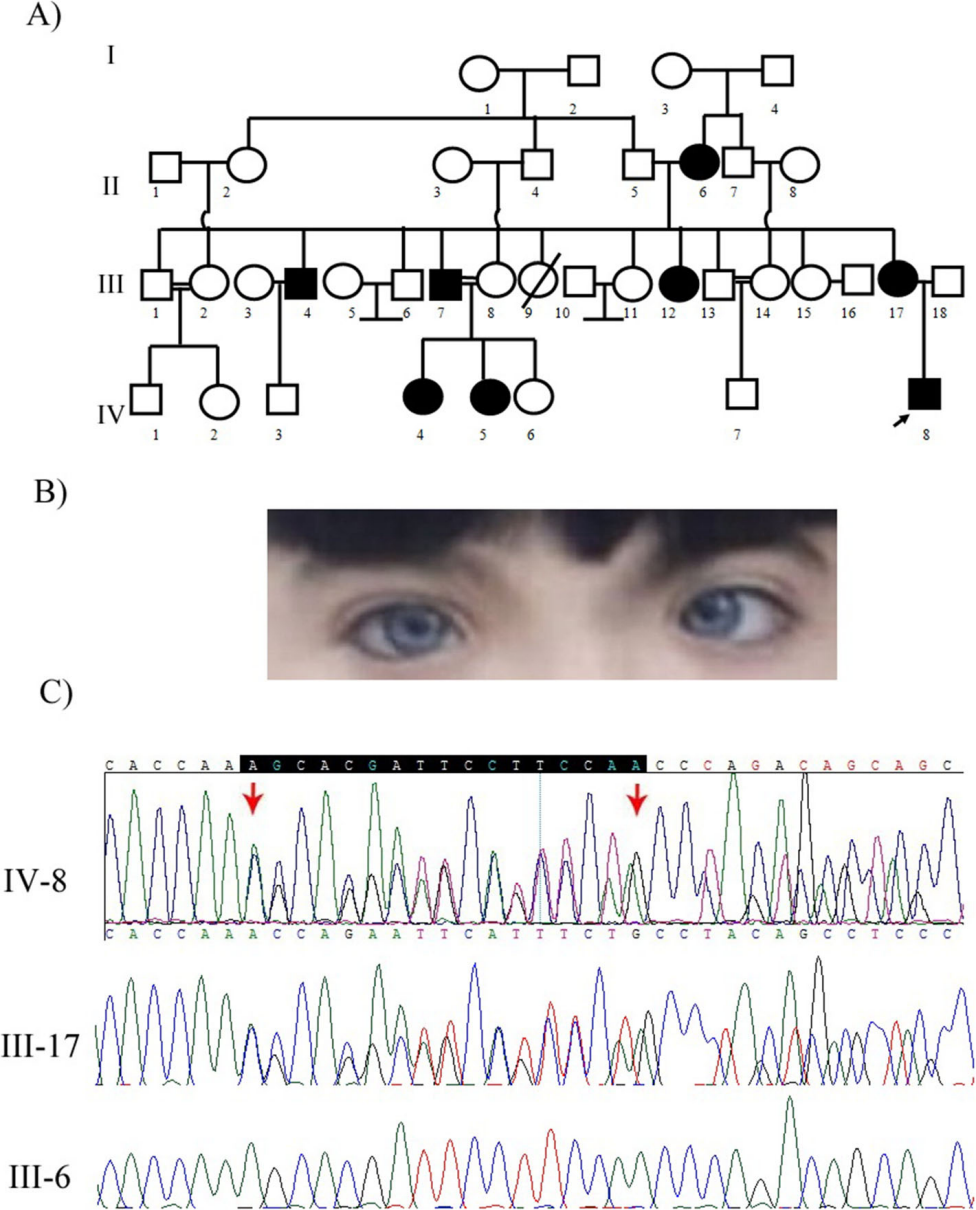
**a** Pedigree of family 4 with Waardenburg syndrome type 1 with *PAX3* mutation. The black arrow indicates the proband (IV-8). **b** Iris color of Waardenburg syndrome type 1 patient. IV-8, brilliant blue irises. **c** The DNA sequence chromatogram of exon 7 of the *PAX3* gene with the frameshift mutation c.1024_1040del AGCACGATTCCTTCCAA (p.S342Pfs*62) in heterozygous state in proband (IV-8) and proband^’^s mother (III-17). This mutation was not detected in proband^’^s uncle [mother-side] (III-6). The black arrow above the chromatogram sequence shows the c.1024_1040del AGCACGATTCCTTCCAA mutation

Four unrelated Iranian families were referred to Rasad Pathobiology and Genetic Laboratory, Tehran, Iran with the chief complaint of congenital hearing loss. A comprehensive clinical history was gathered and clinical examinations were meticulously performed for each affected individual. Our study included four WS patients and nine additional family members **(**Figs. 1, 2, 3 and 4A**)**. The clinical features suggested a diagnosis of WS for the probands. Written informed consent was obtained from the patients’ parents and all participants included in this study.

Family 1: The proband (VI-2) was the second child of a non-consanguineous marriage and he was a 1-year-old symptomatic boy with congenital hearing loss and heterochromia iridis with the blue segment in his left iris. His older sister (VI-1) was also affected by congenital hearing loss and brilliant blue eyes **(**Fig. 1B**)**. His mother presented with a white frontal forelock and skin hypopigmentation on her hands and face. His aunt (V-6) and another relative (V-3) had hearing loss as well. The parents (V-7 and V-8), proband, and his older sister were included in this study.

Family 2: A 1.5-year-old symptomatic girl (III-3) showed a congenital profound hearing loss, brilliant blue eyes, and skin hypopigmentation on the abdominal region at birth but she did not display dystopia canthorum **(**Fig. 2B**)**. Her brother (III-2) was healthy, but her maternal grandfather (I-2) and some relatives had brilliant blue eyes. The patient and her healthy parents (II-2 and II-3) were included in this study.

Family 3: The proband (III-2) was an 8-month-old symptomatic male with brilliant blue eyes, congenital profound hearing loss, developmental delay, hypotonia, skin hypopigmentation on her hand and foot, and Hirschsprung disease. Colostomy was performed to remove the non-functional segment of the intestine at age of 2 years. Brain magnetic resonance imaging (MRI) revealed mild atrophy **(**Fig. 3B**)**. A positive history of hearing loss and polydactyly was detected in the family. The parents were not consanguineous and did not manifest any pertinent clinical features of the disease. The patient and his healthy parents (II-3 and II-4) were included in our study.

Family 4: medical examinations showed that the proband-a 4-year-old symptomatic boy (IV-8)- was suffering from dystopia canthorum, broad nasal root, synophrys, skin hypopigmentation on her hand and abdomen, brilliant blue eyes, and congenital profound hearing loss **(**Fig. 4B**)**. Five affected individuals (II-6, III-4, III-7, III-12, and III-17) exhibited identical phenotypes, but II-6, III-4, III-7, and III-17 had normal hearing. The patients II-6 and III-17did not manifest any pigmentary disturbances of iris. The III-4 and III-7 as the affected individuals presented premature greying before age 30 and heterochromia iridis, respectively. Bilateral congenital hearing loss was evident in IV-4 and IV-5; IV-4 had brilliant blue eyes by the way. There was a history of paralysis and cleft lip in the mother’s relatives. Two affected patients (IV-8 and III-17) and a healthy individual (III-6) were included in our study.

Whole-exome sequencing was performed on the probands’ samples so as to identify the causal genes. To this end, the blood samples were obtained from the proband of each family. Genomic DNA was then extracted from whole blood using standard extraction methods.

Library preparation was performed using the Twist Core Exome kit (TWIST Bioscience, USA) according to the manufacturer’s instructions. Sequencing of the libraries was performed by high-throughput paired-end sequencing using the NovaSeq sequencing platform (Illumina Inc., CA, USA) as a service by CeGaT GmbH, Germany. Alignment to the reference human genome hg19 from UCSC genome browser (University of California, Santa Cruz, USA) was carried out by the Burrows-Wheeler Aligner (BWA) program. Variant calling and filtering were performed using the Genome Analysis Toolkit (GATK-v3.4.0). Detected variants were annotated using Ensembl (https://www.ensembl.org), RefSeq (https://www.ncbi.nlm.nih.gov/refseq/), dbSNP (https://www.ncbi.nlm.nih.gov/snp/), gnomAD *(*https://www.gnomad.broadinstitute.org), 1000 Genomes (https://www.1000genomes.org) and, OMIM (https://www.omim.org) databases.

To narrow down the derived variants, to begin with, the variants located on genes with no reported human disease were removed from the annotated list of variants. In the next step, variants with alternate to total read ratio of < 0.2 were removed. Then, the minor allele frequency of < 0.01 on public databases, e.g. gnomAD and dbSNP, was included as another filter to reduce the number of variants. After applying such filters then variant consequences were used to prioritize remaining variants for further interpretation in terms of pathogenicity based on American College of Medical Genetics and Genomics (ACMG) guideline. Multiple *in silico* tools including Mutation taster, and CADD were used to evaluate the effect of detected variants on gene or gene products. Evolutionary conservation of genomic position was assessed using Phylop and PhastCons scores.

ClinVar (http://www.ncbi.nlm.nih.gov/clinvar/), HGMD (http://www.hgmd.cf.ac.uk/), OMIM, and PubMed were reviewed for previous reports or publications related to pathogenic variants in *MITF* gene.

The mutations detected by whole-exome sequencing were investigated in patients and all the participants by PCR-Sanger sequencing methods using specific primers. The primers used for amplification has been described in Table 1. The PCR mixture was set in a total volume of 25 µl. All PCR amplifications were performed using 12.5 µl PCR master mix (Yekta Tajhiz Azma, Iran), 8.5 µl H_2_O, 1 µl of each primers described in Table 1, and 2 µl extracted DNA. The reaction was started with an initial denaturation at 95˚C for 5 min, followed by 35 cycles at 95˚C for 30 s, annealing at various temperature for 45 s for the different primers, and extension at 72˚C for 45 s, and a final extension at 72˚C for 7 min on a thermal cycler (Veriti, Applied Biosystems, USA). The PCR products were analyzed on 2 % agarose gel and then the products were purified and sequenced on ABI 3500 Genetic analyzer.

**Table 1:** PCR Primers Sequences

Gene exon	Forward primer sequence (5ʹ- 3ʹ)	Reverse primer sequence (5ʹ- 3ʹ)	Product length (bp)
*MITF*-exon8	AGAATTTGGGCTTTCACCAG	CTTGATGAGACTAACCAAAAGAAAG	590
*SOX10*-exon3	CCAGCCCATGAA AGATTTGG	TGCCCCAGCCACCTCTC	1,090
*PAX3*-exon7	GGTGGCTGATGAACTTTTGC	AGAAACACGGGACTGACCTG	313

**Table 2:** Identified variants in this study

Gene/ Transcript (RefSeq)	Variant Location	Variant	Chromosome Position (GRCh37)	Zygosity^a^	OMIM number^b^	Inheritane Pattern^c^	Variant Classification^d^
*MITF* NM_198159.3	Exon 8	c.996delT p.K334Sfs*15	Chr3: 70,005,662	Het	193,510	AD	Pathogenic
*MITF* NM_198159.3	Exon 8	c.950G > A p.R317K	Chr3: 70,005,618	Het	193,510	AD	Likely pathogenic
*SOX10* NM_006941.4	Exon 3	c.684delC p.E229Sfs*57	Chr22: 38,373,887	Het	609,136	AD	pathogenic
*PAX3* NM_181459.4	Exon 7	c.1024_1040delAGCACGATTCCTTCCAA p.S342Pfs*62	Chr2: 223,084,992	Het	193,500	AD	Pathogenic

^a^*Het *Heterozygous, ^b^*OMIM number *Five-digit number assigned to each phenotype in Online Mendelian Inheritance in Man (OMIM) database, ^c^*AD *Autosomal dominant. ^d^Based on American College of Medical Genetics and Genomics (ACMG) standards and guidelines for the interpretation of sequence variants, 2015

* = termination codon

In this study, we report four distinct patients of WS in the Iranian population. Using whole-exome sequencing, a frameshift deletion, c.996delT (p.K334Sfs*15), was found in exon 8 of the *MITF* gene in the first patient (VI-2). The same mutation was validated in the patient, her affected mother (V-7), and sister (VI-1) in a heterozygous state by PCR and Sanger sequencing methods, but was not detected in his asymptomatic father (V-8) **(**Fig. 1 C**)**. Regarding the second patient (III-3), the mutation, c.950G > A (p.R317K), on exon 8 of the *MITF* was detected by whole-exome sequencing and confirmed by PCR and Sanger sequencing methods in the patient. This mutation was *de novo* and was not detected in her unaffected parents (II-2 and II-3) with the Sanger sequencing method **(**Fig. 2 C**)**. In the third patient (III-2), a frameshift deletion, c.684delC (p.E229Sfs*57), in exon 3 of the *SOX10* gene was identified in the heterozygous state. However, parental DNA analysis (II-3 and II-4) did not detect any mutation, underscoring that this mutation was *de novo***(**Fig. 3 C**)**. In the last case (IV-8), a pathogenic frameshift mutation, c.1024_1040del AGCACGATTCCTTCCAA (p.S342Pfs*62) on exon 7 of the *PAX3* gene was detected as a heterozygous mutation. The mutation was co-segregated in all available affected family members **(**Fig. 4 C**) (**Table 2**)**.

All detected mutations were analyzed using *in silico* tools, e.g. MutationTaster and CADD. Available databases were used to review the reported pathogenic variants in *MITF, SOX10*, and *PAX3* genes. All variants were classified as the “pathogenic variants” based on ACMG guidelines.

## Discussion and conclusions

In this report, we describe the clinical features of four genotyped unrelated cases with frameshift deletions in the *MITF*, *SOX10*, and *PAX3* genes and a missense mutation in the basic domain of the *MITF* gene.

WS2A -a subset of WS2- is an autosomal dominant disorder characterized by variable degrees of sensorineural hearing loss and pigmentary disturbances of skin, hair, and eyes. Heterozygous mutations in the *MITF* gene cause WS2A and lack of normal melanocytes in the affected organs, e.g. eye, skin, and the cochlea [32–34]. In the patient of family 1, a heterozygous novel mutation, c.996delT (p.K334Sfs*15), was identified in exon 8 of the *MITF*. This mutation was a single-base deletion of a T at base 996 within the loop of the MITF protein, resulting in a frameshift and premature stop codon at 15 codons downstream from the deletion point. Therefore, it is truncated at the second helix portion lacking twelve residues of the second helix and the complete leucine zipper region. This mutation results in a truncated MITF protein with 348 amino acids instead of the wild type length of 520 amino acids. Therefore, about 33 % of the protein length has been deleted [35, 36]. No dystopia canthorum, musculoskeletal abnormalities, or Hirschsprung disease was observed in affected members of this family and the pedigree chart showed autosomal dominant inheritance. Based on Waardenburg Consortium, clinical manifestations and the detected variant in the *MITF* gene in our patients, diagnosis of WS2A was confirmed [6]. Multiple lines of *in silico* computational analysis (e.g. CADD, Mutation Taster, etc.) supported the deleterious effect of the variant. The bHLHZip structure of MITF has an important role in its function and an important role of the HLHZip is in dimerization. The mutation found in MITF seems that disrupted the bHLHZip domain and impaired the function of MITF protein and it is thought to have defects in homo- or heterodimerization and DNA binding. As a result, this can affect the expression of its target genes which leads to the absence of normal melanocytes in the skin, eye, and the cochlea [34, 37]. Previous studies indicated that the stop mutation p.R255^*^ and p.R259* in the *MITF* exon 8 in patients with WS2A resulted in truncated MITF proteins in the second helix domain, thus, lost their DNA binding ability and homo- or heterodimerization activity and also p.R259* failed to activate expression of the *TYR*, *TYRP1*, and *DCT* genes. Also, Yan et al. in 2011 reported a heterozygous mutation c.742_743delAAinsT;746_747delCA in exon 8 of the *MITF* gene in a large Chinese family with WS2A that this mutation resulted in a premature termination codon within the second helix of MITF protein. Therefore, a non-functional MITF protein was produced due to loss of the bHLHZip structural domain and was led to haploinsufficiency [12, 35, 38, 39].

The microphthalmia (MiT) family of transcription factors includes MITF, TFEB, TFEC and TFE3 which share a common basic-helix-loop-helix-leucine zipper (bHLHZip) dimerization motif and a transactivation domain (TAD). Nine different MITF isoforms have been produced from a single *MITF* gene and all isoforms have exons 2–9 in common [36]. MITF is expressed in melanocytes and contains a basic domain for DNA binding and an HLHZip domain for homo- or heterodimerization with either TFEB, TFEC, or TFE3, which recognizes specific DNA sequences of pigmentation genes, including tyrosinase (TYR), tyrosinase-related protein 1 (TRP1) and DCT/TRP2 that are responsible for melanogenesis and regulates the expression of these genes; therefore, it has an important role in melanocytes development and differentiation. MITF isoform A is composed of 9 exons, which consists of 520 amino acid residues. This transcription factor is expressed in many cell types including melanocytes and retinal pigment epithelium (RPE) cells [12, 35, 37, 40–42].

A previous study demonstrated that mutations of Ser298 in MITF-M (Ser399 in MITF-A), which is located downstream of bHLHZip, has an important role in the transcriptional function of MITF. It has been shown that phosphorylation of Ser298 by Glycogen synthase kinase 3 (GSK3) enhances the ability of MITF to bind the DNA *in vitro*. Consequently, the phosphorylation of MITF by GSK3β in this truncated protein is abolished impairs the function of MITF that can be a potential cause of WS2. Also, serine-rich and threonine-rich regions of MITF are important for its function. Thus, transactivation assays revealed that truncation of the last 95 amino acids (including threonine- and serine-rich regions) decreased the transcriptional activity of MITF (MITF-M[1-324], MITF-A[1-425]. Transactivation assays and DNA-binding assays revealed that deletion of (MITF-M[294–324], MITF-A[395–425]) which is downstream of the bHLHZip structure, decreased the transactivation activity and ability of MITF to bind to the E-box, respectively. As a result, the truncated mutation -p.(K334Sfs*15)- does not have the last 95 amino acids and also the amino acids from 395 to 425 that can affect its transcriptional activity, transactivation capacity, and DNA binding ability [35, 37]. The mutation described in this study results in a premature translational termination codon in the last translated exon, thus, it is predicted that induce escaping the mutant transcript from the nonsense-mediated mRNA decay (NMD) pathway [11, 19]. Nobukuni et al., showed that the MITF proteins with truncated mutations within the bHLHZip region lost their ability to dimerize with wild-type MITF proteins, but they did not have any interfering with DNA binding activity of wild-type MITF protein, therefore, the clinical characteristics of patients with WS2A are caused by loss of function mutations resulting in haploinsufficiency of the MITF protein. Haploinsufficiency appears to be a major mechanism in deafness and pigmentary abnormalities of WS2 patients [34, 37]. Different kinds of mutations have been reported in *MITF* such as missense, nonsense, and splicing mutations [43].

A missense mutation, c.950G > A (p.R317K), in the MITF-A, corresponding to p.Arg216Lys in the MITF-M (GenBank: NM_000248.1), was identified in exon 8 of the *MITF* in the second patient with WS2A and resulted in an amino acid arginine being replaced by lysine at residue 317. This novel and non-truncating variant is located at the basic domain of the MITF protein [35]. Therefore, it would be expected to impair the DNA binding ability of MITF protein, and it is thought to be responsible for the disease. The proband’s parents did not carry the same mutation indicating that the mutation occurred *de novo*. Tietz syndrome (TS; OMIM: 103,500) is a more severe form of WS2A and it is characterized by profound congenital deafness and generalized hypopigmentation of skin, hair, and eye that is transmitted in an autosomal dominant manner with heterozygous mutations within the *MITF* gene, while WS2A is differentiated from Tietz syndrome by patchy depigmentation abnormalities of the skin, hair, and irides [32, 33]. Our patients with *MITF* mutations had a less severe phenotype without generalized hypopigmentation of skin, hair, and eye. A TS-causing mutation of p.Arg216Ser and a *de novo* missense mutation p.Arg216Lys, which are located at the basic domain of the MITF protein, have been previously reported. Although these two mutations are located at the same amino acid position, Arg216Ser caused a more severe phenotype than a missense mutation p.Arg216Lys, thus the type of amino acid in the location of the mutation in the protein can be an important factor in the determination of TS and WS2A phenotype. Also, WS2A-associated p.Arg216Lys was demonstrated to lack DNA binding and activate expression from the *TYR*, *TYRP1*, and *DCT* promoters. Arg216 is a conserved residue in MITF proteins among different species [32, 35, 38]. *De novo* missense mutations p.Glu213Asp, p.Arg216Lys, p.Arg217Gly, and p.Arg217Ile, in patients with hearing loss and blue irides, located at the basic domain of the MITF protein have been previously reported associated with WS2A and failed to bind DNA and activate expression of the *TYR*, *TYRP1*, and *DCT* genes [12, 23, 38, 44]. Also, heterozygous mutations p.Lys307Asn, p.Leu312fs*11, p.Δ315Arg (c.944_946delGAA), p.Arg318Gly and p.Arg318del within the basic domain of MITF-A has been reported to cause WS2A [8, 35, 45]. According to the Leiden Open Variation Database (http://grenada.lumc.nl/LOVD2/WS/), the majority of *MITF* mutations are located in exons 7, 8, and 9 that encode the bHLH-zip domain [3, 8]. This variant is predicted to be damaging by *in silico* analysis using SIFT.

Regarding patient 3, a *SOX10* heterozygous mutation, c.684delC (p.Glu229Serfs*57), was identified. This *SOX10* frameshift mutation is proximal to the 3’-end of exon 3 and is localized at the intervening sequence of the HMG domain and K2 region which shifts the reading frame after codon 229, resulting in a premature termination codon within the K2 conserved region of the SOX10 protein. Therefore, the truncated SOX10 protein has 285 amino acids instead of the wild type length of 466 amino acids [22]. SOX10 is a member of the SOX family of transcription factors involved in the development of the enteric nervous system (ENS), melanocytes, glial cells, and intestinal ganglia cells. It has 4/5 exons with three coding exons which encode a protein of 466 amino acids. SOX10 possesses a dimerization domain (AA61-101), a DNA-binding high-mobility group (HMG) domain (101–180), a conserved domain in the center of the protein (233–306), and a transactivation domain (400–466) at the C-terminus. Mutations in *SOX10* are associated with WS2E and WS4C (OMIM: 613,266), while mutations in *SOX10* that are responsible for WS4 with neurological features referred to as PCWH (OMIM: 609,136) which patients may suffer from peripheral neuropathy, mental retardation, cerebellar ataxia, neonatal hypotonia and spasticity [5, 14, 46–48]. PCWH is mostly caused by truncating mutations in the last coding exon of *SOX10* or less than 50–55 nucleotides upstream of the last coding exon, induce escaping the mutant mRNAs from the NMD pathway. Thus, the synthesized mutant protein act as a dominant-negative protein that interferes with the activity of the wild-type SOX10, resulting in neurological symptoms in WS. The previous report showed that a WS patient with a truncating mutation of c.652G > T, p.G218* in exon 3 of the *SOX10* had neurological symptoms [47]. SOX10 indeed interacts with PAX3 and regulates the MITF expression, so it can be hypothesized that SOX10 or PAX3 dysfunction impairs MITF expression. Also, *in vitro* studies showed that the S251X mutation within the conserved domain of SOX10 abolishes The SOX10-dependent activation as well as synergistic activation of the MITF promoter with PAX3. The conserved region in SOX10 has a crucial role in mediating the synergy between SOX10 and PAX3; hence, axiomatically, any truncating mutations in this region can impair the ability of SOX10 to interact with PAX3 [5, 10]. Consistent with the previous study it is predicted that the truncating mutation c.684delC (p.Glu229SerfsTer57) identified in this study loses its ability to activate MITF promoter and its interaction with PAX3. This mutation results in a premature translational termination codon in the last coding exon of SOX10 that escapes the NMD pathway and act as a dominant-negative protein that interferes with the activity of the wild-type SOX10. This result underscored that the clinical manifestations of this patient were in line with this mutation [22]. The *SOX10* mutations associated with the classical form of WS4 and PCWH are present heterozygously and most often occur *de novo* [5]. The analysis of the unaffected parents’DNA by PCR-Sanger sequencing revealed the *de novo* occurrence of the mutation. Multiple lines of *in silico* computational analysis (CADD, Mutation Taster, etc.) supported the deleterious effect of the variant. The majority of the *SOX10* mutations are embracing the truncating mutations such as nonsense and frameshift. The other types of mutations comprise splice mutations, in-frame insertions, substitution, and partial or full gene deletions [5, 49]. Previous studies have reported some PCWH-related mutations including p.Gln234X, p.Gln250X, p.Ser251X, p.Gly266AlafsX20, and p.His283LeufsX11 in SOX10 conserved domain [28].

In the present study, the WES revealed a heterozygous mutation c.1024_1040del AGCACGATT CCTTCCAA (p.Ser342ProfsX62) in exon 7 of the *PAX3* gene in the fourth patient with dystopia canthorum, a typical characteristic of WS1, which has been reported previously. This 17-base deletion occurred within the TAD of the *PAX3* gene and altered the reading-frame followed by premature translational of a stop codon in TAD. This truncating mutation can lead to a truncated PAX3 protein with 403 amino acids instead of the wild type length of 505 amino acids, it has functional paired domain and homeodomain but a loss of a part of TAD would be expected to affect its normal function. PAX3 encodes a member of the paired box of transcription factors that is involved in the development of the central nervous system, skeletal muscle, and melanocytes. There are eight human PAX3 isoforms, among which, PAX3e is the longest isoform consists of 10 exons and encodes a paired domain (PD) by exons 2–4, a homeodomain (HD) by exons 5 and 6, and a Ser-Thr-Pro-rich C terminus transactivation domain (TA) by exons 6–8. The PD and HD are DNA binding domains, while the TA domain mediates transcriptional regulation. The translated regions encode a 505-amino acid protein [5, 19, 48, 50, 51]. A previous functional study has demonstrated that the truncating mutation 1185 insTGA which removes a part of the transactivation domain of PAX3 reduced its effect on MITF promoter activation, and also its synergistic effect with SOX10 was completely lost. As a result, it would be expected that the c.1024_1040del AGCACGATTCCTTCCAA mutation found in this study causes losing its ability to activate MITF promoter and its interaction with SOX10 [10]. WS1 is caused by loss of function mutations in the *PAX3* gene leading to haploinsufficiency [34, 52].

All WS clinical features show variable expressivity among the affected individuals [4, 11, 39]. Therefore, there was considerable variability in phenotypes of members of this family. We found that 5 out of 8 (62.5 %) affected members of family four had iris pigmentary disturbances including brilliant blue eyes and complete heterochromia iridis. Four affected individuals (50 %) had hearing loss while other patients had normal hearing. Dystopia canthorum, broad nasal root, synophrys, and skin hypopigmentation were observed in 6 patients (75 %) of this family. No musculoskeletal abnormalities were detected in each patient of the family. The father (III-18) was healthy and there was not any phenotype of the disease in anybody of the next of the kins. Thus, analysis of his affected mother’s DNA by PCR-Sanger sequencing revealed that she was the carrier of the same heterozygous mutation but failed to demonstrate the mutation in his healthy uncle (III-6). According to Waardenburg Consortium, clinical features of our patient and the genetic mutation in the *PAX3*, he was classified into the WS1 group [7]; the pedigree analysis showed that the variant was segregated with WS phenotypes as an autosomal dominant trait in this family. Most patients of WS1 manifest heterozygous mutations in the *PAX3* gene [53]. Different *PAX3* mutations including missense, nonsense, frameshifts, small in-frame indels, and splice alterations have been reported in WS1 patients; for instance, some WS1-related mutations have been reported that in turn include p.Gly292ArgfsX118, p.Phe294ValfsX116, p.Pro318HisfsX63, and p.Leu396X in the PAX3 transactivation domain [5, 31].

Based on the ACMG criteria [54], the c.996delT variants of *MITF*, c.684delC of *SOX10*, and c.1024_1040del AGCACGATTCCTTCCAA of *PAX3* were considered to be pathogenic and c.950G > A of *MITF* is likely pathogenic. The three reported variants c.996delT, c.950G > A, and c.684delC in this study are absent in population databases such as Exome Aggregation Consortium (ExAC) (http://exac.broadinstitute.org/), 1000 Genomes (http://www.1000genomes.org/), dbSNP (http://www.ncbi.nlm.nih.gov/snp/) and our local database.

In conclusion, we identified three novel as well as a previously reported mutation in *MITF*, *SOX10*, and *PAX3* gene in four unrelated Iranian families. Three mutations reported in this study including c.996delT (p.K334Sfs*15) in exon 8 of the *MITF*, c.684delC (p.Glu229Serfs*57) in exon 3 of the *SOX10* and c.1024_1040del AGCACGATTCCTTCCAA (p.Ser342Profs*62) in exon 7 of the *PAX3* gene are frameshift and truncating mutations which ultimately results in early termination, leading to the formation of a truncated protein that can affect their normal function and a missense mutation reported in this study impairs DNA binding ability of MITF protein. Functional analysis is required to explore the exact mechanisms of pathogenesis of these mutations. An exact description of the mutations responsible for WS provides useful information to explain the molecular cause of clinical features of WS and contribute to better genetic counseling of WS patients and their families. Also, our study expands the mutation spectrum of *MITF*, *SOX10*, and *PAX3* genes.

## Data Availability

The datasets used and/or analyzed during the current study are available from the corresponding author on reasonable request.

## Abbreviations

WS: Waardenburg syndrome
PAX3: Paired box gene 3
MITF: Microphthalmia-associated transcription factor
SOX10: SRY (sex-determining region Y)-box10
SNAI2: Snail homolog of 2
EDNRB: Endothelin receptor type B
EDN3: Endothelin-3
WES: Whole-exome sequencing
MRI: Magnetic resonance imaging
MiT: Microphthalmia
bHLHZip: Basic-helix-loop-helix-leucine zipper
TAD: Transactivation domain
TYR: Tyrosinase
TRP1: Tyrosinase-related protein 1
RPE: Retinal pigment epithelium
GSK3: Glycogen synthase kinase 3
NMD: Nonsense-mediated mRNA decay
TS: Tietz syndrome
ENS: The enteric nervous system
HMG: High-mobility group
PCWH: Peripheral demyelinating neuropathy, Central dysmyelinating leukodystrophy, Waardenburg syndrome, Hirschsprung disease
PD: Paired domain
HD: Homeodomain
